# User Experience of an App-Based Treatment for Stress Urinary Incontinence: Qualitative Interview Study

**DOI:** 10.2196/11296

**Published:** 2019-03-14

**Authors:** Ina Asklund, Eva Samuelsson, Katarina Hamberg, Göran Umefjord, Malin Sjöström

**Affiliations:** 1 Department of Public Health and Clinical Medicine, Family Medicine Umeå University Umeå Sweden

**Keywords:** mobile applications, urinary incontinence, stress, pelvic floor muscle training, self-management, qualitative research, grounded theory, women’s health

## Abstract

**Background:**

Stress urinary incontinence (SUI) affects 10%-39% of women. Its first-line treatment consists of lifestyle interventions and pelvic floor muscle training (PFMT), which can be performed supervised or unsupervised. Health apps are increasing in number and can be used to improve adherence to treatments. We developed the Tät app, which provides a 3-month treatment program with a focus on PFMT for women with SUI. The app treatment was evaluated in a randomized controlled trial, which demonstrated efficacy for improving incontinence symptoms and quality of life. In this qualitative interview study, we investigated participant experiences of the app-based treatment.

**Objective:**

This study aimed to explore women’s experiences of using an app-based treatment program for SUI.

**Methods:**

This qualitative study is based on telephone interviews with 15 selected women, with a mean age of 47 years, who had used the app in the previous randomized controlled trial. A semistructured interview guide with open-ended questions was used, and the interviews were transcribed verbatim. Data were analyzed according to the grounded theory.

**Results:**

The results were grouped into three categories: “Something new!” “Keeping motivation up!” and “Good enough?” A core category, “Enabling my independence,” was identified. The participants appreciated having a new and modern way to access a treatment program for SUI. The use of new technology seemed to make incontinence treatment feel more prioritized and less embarrassing for the subjects. The closeness to their mobile phone and app features like reminders and visual graphs helped support and motivate the women to carry through the PFMT. The participants felt confident that they could perform the treatment program on their own, even though they expressed some uncertainty about whether they were doing the pelvic floor muscle contractions correctly. They felt that the app-based treatment increased their self-confidence and enabled them to take responsibility for their treatment.

**Conclusions:**

Use of the app-based treatment program for SUI empowered the women in this study and helped them self-manage their incontinence treatment. They appreciated the app as a new tool for supporting their motivation to carry through a slightly challenging PFMT program.

**Trial Registration:**

ClinicalTrials.gov NCT01848938; https://clinicaltrials.gov/ct2/show/NCT01848938 (Archived by WebCite at https://clinicaltrials.gov/ct2/show/NCT01848938)

## Introduction

Urinary incontinence (UI) is defined as complaints of any involuntary leakage of urine [1]. There are different types of UI, the most common of which is stress urinary incontinence (SUI). SUI is defined as leakage of urine with exercise, coughing, or sneezing and affects 10%-39% of women [1-3]. Although common, many women do not seek care because they regard their incontinence as “normal” or find it too embarrassing to talk about [4]. The first-line treatment of SUI consists of lifestyle interventions and pelvic floor muscle training (PFMT) and can be initiated among most women without extensive preliminary evaluation [5]. A recent Cochrane review confirms that PFMT is effective and can cure or improve symptoms of SUI, reduce the number of leakage episodes, and improve UI-specific quality of life [6]. There is no consensus on the optimal PFMT program, but it usually involves exercises designed to increase muscle strength, endurance, rapidity, and coordination [7]. Adherence to the exercise program is important for effectiveness, and strategies to increase adherence need more attention among clinicians and researchers [8].

Smartphone use is increasing worldwide, and in 2017, more than 32% of the world’s population used a smartphone [9]. In Sweden, 85% of the population owned a smartphone in 2017, and the corresponding number in the United States was 77% in 2018 [10-11]. The number of health apps is increasing rapidly, with 325,000 mobile health apps available in 2017 [12]. Health apps have the potential to increase treatment adherence and improve clinical outcomes in chronic disease management [13]; however, data about user experience with health apps are limited, because many qualitative studies focused on the developmental stage of the apps, not experiences with long-term use [14].

Within the eContinence project [15], the Tät app was developed by ES, MS, and GU in cooperation with engineers at ITS (ICT Services and System Development), Umeå University, Sweden. The app, which provides a treatment program for SUI with a focus on PFMT, is referred to here as the “app-based treatment.” We demonstrated efficacy regarding symptoms, quality of life, and urinary leakage in a previous randomized controlled trial (RCT) [16]. The treatment was cost effective [17] and had persistent long-term effects [18].

As part of the evaluation of the app-based treatment, we aimed to learn more about the participants’ experiences of using the app and engaging in the PFMT program. We wanted to determine how and why the app-based treatment could be effective. The aim of this study was therefore to explore women’s experiences of using an app-based treatment program for SUI.

## Methods

### Study Setting and Design

This qualitative study was based on telephone interviews in Sweden between January and November 2014. All participants had taken part in our previous RCT (Trial Registration: ClinicalTrials.gov NCT01848938) [16] that evaluated the effect of the mobile app Tät. We received ethical approval from the Regional Ethical Review Board of Umeå University (UMU DNR: 2012-325-31M).

For the RCT, we recruited adult women who experienced SUI at least once a week through our website. Exclusion criteria were pregnancy, previous urinary incontinence surgery, malignancy in the lower abdomen, neurologic disease affecting the lower abdomen or legs, irregular bleeding, visible blood in urine, difficulty passing urine, and severe psychiatric disorders. Participants completed an informed consent form, a Web-based questionnaire, and a 2-day leakage diary before randomization. We randomized 123 women to treatment with the app for 3 months (n=62) or a postponed treatment group that did not receive the app until the follow-up was completed (control group, n=61). During the whole RCT study, from online recruitment to follow-up, we had no face-to-face contact with any of the women in any of the study groups. We did not provide any reimbursements. At 3 months of follow-up, we evaluated the effect of the app-based treatment with Web-based questionnaires. The methods and results from the RCT are presented in detail elsewhere [16].

**Figure 1:**
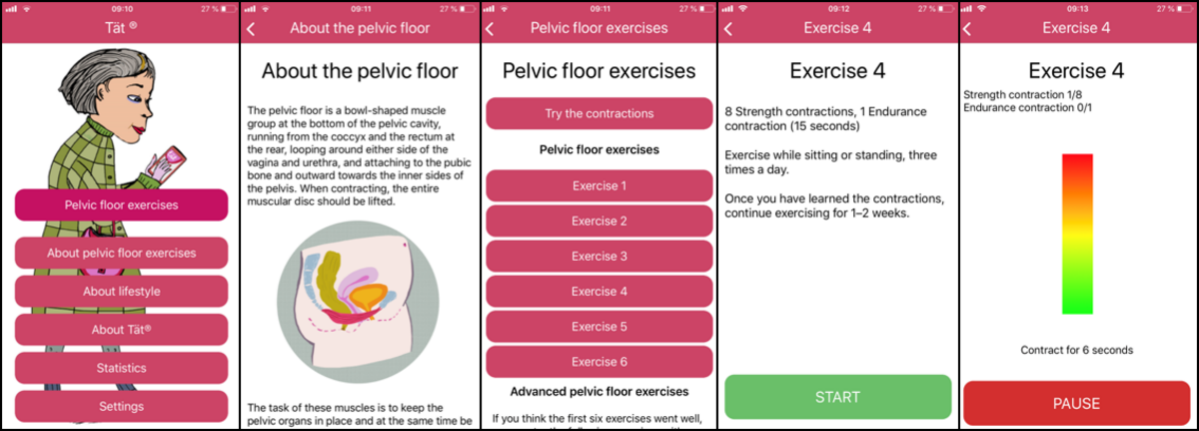
Screenshots of the mobile app Tät version 1.9.

The app included a treatment program focused on PFMT exercises and contained information about SUI; the pelvic floor; and factors related to incontinence such as overweight, physical activity, smoking, and fluid intake. There were six basic and six advanced PFMT exercises with increasing difficulty, and the participants were advised to proceed according to their own improvement. The exercises included different types of pelvic floor muscle contractions such as quick, strength, and endurance contractions. To illustrate each contraction, a moving graph showed how long and with what intensity the user should contract and relax (Figure 1). Within the app, the participants could set three reminders a day and log the number of exercises performed in a statistics function.

### Participants

**Figure 2:**
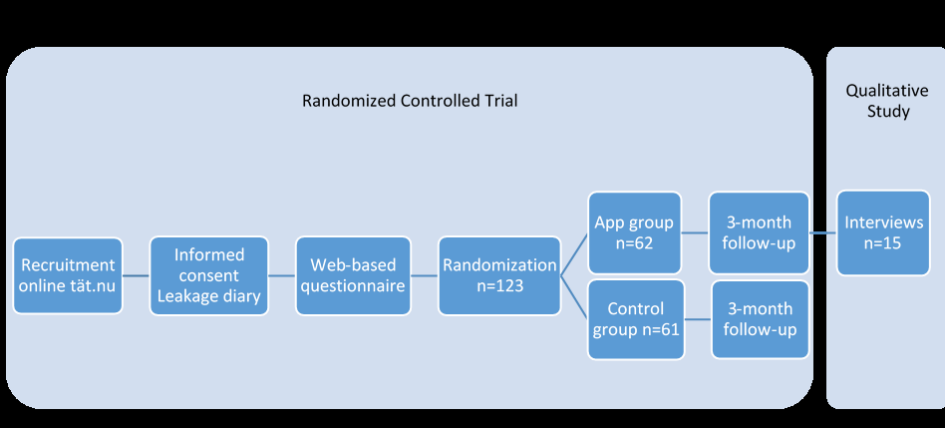
Flow of the randomized controlled study and the present interview study.

Participants eligible for this interview study were women from the RCT app group (who received the app directly after randomization) from our previous RCT, who had completed the 3-month follow-up. The first author IA performed the selection and approached the participants by email invitation. Our aim was to conduct the interviews within 3 months after they had completed the follow-up in the RCT in order to minimize the risk of recall bias. In Figure 2, we summarize the flow of the previous RCT study and the present interview study.

We purposefully selected participants with a goal of ensuring diversity of age, residence, and type of mobile phone operating system (Apple or Android). For the last interviews, we also selected two participants based on their treatment outcome in the RCT, because we wanted experiences from women who did not show improvement in incontinence symptoms.

### Data Collection

Individual telephone interviews were conducted using a semistructured interview guide with open-ended questions. The first author developed the interview guide, which was discussed in the research group to refine the questions. To determine which topics could be worth investigating further, we used information from the participants’ answers to open-ended questions about their experiences of the app-based treatment in the follow-up questionnaire from the RCT. Some of the research team also had previous experience in internet-based treatment of incontinence, which influenced our preunderstanding.

The topics covered in the interviews were as follows: the participants’ expectations about using an app for treatment, their experiences of using the app, their relationship and interaction with the app during the study, and their experiences performing PFMT independently.

The first author IA, who is a general practitioner, conducted all interviews. As suggested for qualitative analyses, we performed data collection, transcription, and preliminary analysis in parallel [19,20]. This approach made it possible to adapt the interview guide when new, interesting aspects emerged. For example, we added questions to explore in more detail how the participants interacted with the app. We also added questions about the participants’ expectations for the app and their views on PFMT, in general.

Interviews were recorded and transcribed verbatim. The first author transcribed 8 of the 15 interviews, and an experienced medical secretary transcribed the remaining 7 interviews. The interviewer wrote short memos after the interviews.

### Data Analysis

We performed the analysis according to the constant comparison techniques in the grounded theory [20]. Our goal was to provide an informative, rich description rather than develop a theory. The analysis started directly after the first interview. All authors independently read and coded the second and third interviews according to meaning and content. The codes were then discussed and sorted into different categories in a joint session. Most of the categories were developed at this stage. Interviews 4 to 10 were coded individually by the first and last authors (IA and MS). The last five interviews were coded only by the first author but the last author read them thoroughly, following which, no new categories emerged.

**Table 1:** Examples of codes, subcategories, and categories derived from a quotation.

Quotation	Code	Subcategory	Category
*That was what was so good about this, I can do this at home myself, no need to book an appointment, find the time and suit others.*	Do it at home Do it myself Having to book appointments Finding the time	Easily accessible Low threshold	Something new!
*Well, to get it sorted, get a regular reminder, get the support day-in, day-out.*	To get it sorted Reminders Daily support	Motivational support	Keeping motivation up!
*But I feel like I have found my muscles, but that I’m still a little bit uncertain about it, kind of.*	Feel I have found my muscles Still a bit uncertain	Insecurity about training correctly	Good enough?

After these steps, all authors read all interviews and met to discuss the categories in relation to the original research questions and see if the data were sufficient to answer our questions. After two additional meetings, we reached conclusions about the categories. The first author reread all interviews to check for similarities and differences in the experiences described. This step was also a way to see that the categories were well grounded in the data and that there were quotations that supported the findings (Table 1). We assumed that we had reached saturation and included a sufficient number of participants when we did not find any new categories in the analysis and the categories we had found were well developed.

## Results

### Principal Findings

Fifteen women between the ages of 27 and 72 years were interviewed (mean age, 47 years). They lived in different parts of Sweden—rural areas, smaller towns, and larger cities. Ten of the participants used iPhones and five used Android operating systems on their mobile phones. The mean interview time was 34 minutes (range, 22-55 minutes). Most of the interviews took place within 3 months after participants completed the follow-up questionnaire in the RCT. Two interviews were delayed until 4 months after follow-up.

At the beginning of the interviews, the participants spoke about their incontinence problems as they were when the women entered the study. Many of them described difficulties when exercising as the biggest problem, followed by problems when playing with their children or coughing or sneezing. There was an ambivalence regarding how they viewed the severity of their incontinence: Although they described it as annoying and affecting their everyday lives, they did not call it a big problem or something that restricted their lives.

Most participants had used apps for other purposes such as bank transactions, social media, calendars, and training and had good experiences. They found this technology uncomplicated and useful in their daily lives.

The analysis resulted in a core category called “Enabling my independence.” The underlying three categories were called “Something new!” “Keeping motivation up!” and “Good enough?” and were each supported by several subcategories (Textbox 1).

### Enabling My Independence

Participants in our study seemed to be health conscious and self-confident, because many of them were actively looking for a self-management program to treat their incontinence. The core category “Enabling my independence” refers to their wish to be independent while needing some external support (like an app) to make it possible. Although disappointed with previous advice and lack of support from health care, the women realized that the only ones responsible for actually performing the PFMT were themselves. They had a strong sense of their own responsibility and felt confident that they could perform the training themselves. The participants were satisfied with trying an app as a tool to support their training and felt that the app helped them realize their wish to follow a PFMT program. It made them feel confident and empowered to know that they had “done what they could” by themselves to treat their incontinence. Although some women expressed a wish for more personalized support, they prioritized the fact that they could handle the treatment independently.

### Something New!

#### New technology, Something New and Modern

Many of the women had searched the internet for new treatment programs and apps addressing PFMT. They had felt that an app could be a suitable tool to use for treatment of incontinence:

And then I thought, maybe there’s an app, there must be an app that helps me to do my pelvic floor exercises, to remind me to do it, because it was mostly the reminder I needed...

The participants perceived the app as a new and modern way of helping them with their incontinence and described their excitement and curiosity about trying it once they had found it. They appreciated that there was an evolution of treatment methods for this condition and that someone was doing research on it. It made them feel that incontinence was an important and prioritized problem:

Yes, great, solve it in a good, I mean use technology in a good way to solve a typically feminine problem, which otherwise is just a scrappy piece of paper from the healthcare centre, you know.

**Categories and subcategories under the core category “Enabling my Independence.”**

**Something new!**

- New technology, something new and modern
- Low threshold, easily accessible

**Keeping motivation up!**

- Motivational support
- Visualizing treatment

**Good enough?**

- Unsure expectations of the result
- Insecurity about training correctly

This feeling was in contrast with their previous experiences with health care in which they felt that their incontinence had been dismissed as a banal problem. They described treatment programs that they had received as old, black-and-white photocopies that were uninspiring and easily forgotten. One woman felt that the advice she received was ignorant and old fashioned:

And then to get a poor photocopy, you know, one of those kind of cut-away views of the genital area. And then just, yes well, it’s also important for you to exercise. And then, well I’ve tried to exercise and it didn’t go well, and then the next person I meet says, like, yes, well, you just have to get used to this being the way it is. Kind of despondent.

It seemed like a treatment program delivered via an app was appealing, as much for its form—a new, modern, and “fun” tool—as for anything else. Some participants also felt that it was easier to talk to friends about a new app for PFMT than to talk about incontinence.

#### Low Threshold, Easily Accessible

One main advantage with following a treatment program in an app seemed to be the proximity to the app and the mobile phone, making the treatment program easily available and convenient to use:

As I use my phone every day and it feels so accessible, well I thought, of course, I always have it with me, so it's easy to find a moment to do the exercises.

The participants appreciated that this treatment was easily accessible from home and was less time consuming than booking appointments in conventional health care:

That was what was so good about this, I can do this at home myself, no need to book an appointment, find the time and suit others, and you know, that process of booking a time.

The participants saw the app treatment as a “first-line” treatment, something to try first, before eventually looking for other help, if necessary. However, the threshold for seeking help in ordinary health care seemed high, and for most women, it was not an alternative at the time.

### Keeping Motivation Up!

#### Motivational Support

Our participants described many barriers to achieving regular PFMT, the most common of which was problems prioritizing training, not being motivated enough, or forgetting to practice:

Well, to get it sorted, get a regular reminder, get the support day-in, day-out. Because there’s so much other stuff that, well you know, life just goes on as it does, and then you end up never doing anything about it because it feels a bit uncomfortable.

The possibility of receiving reminders was the main reason participants had chosen to try an app, and they were convinced that reminders were what they needed to incorporate the routine of training into their daily lives. Most women used the reminder function and described how they tried to perform the exercises when planned, although they sometimes had to postpone the reminders because they were busy at work:

Of course, I’ve read all the brochures and I’ve searched on the internet for pelvic-floor exercises, but you just forget all about it, so what I wanted help with was exactly this, to remember it more.

Participants not using the reminders stated that just seeing the app on the mobile screen reminded them to do the exercises, and one woman even described it as having a person next to her, reminding her to perform the exercises:

It’s works pretty much like when somebody tells you, you know, that you have to exercise.

Some of the participants did, however, describe difficulties in maintaining the same enthusiasm and motivation in the long run. They reported that when the novelty of the app wore off, the training program felt boring. In addition, they reported that in the beginning, they were eager to follow each reminder directly, but at the end of the 3 months, they sometimes ignored the reminders or became annoyed with them. One woman described it somewhat dramatically:

So, during the first month I thought it was kind of fun, because it was new. In the second month I thought like this; oh no...that phone is ringing again, time for me to do my exercises, God how boring. And then during the last month I really had to, pretty much, force myself to do it. But I thought it was so boring.

Another woman said that when her incontinence improved, she lost some of her motivation to do the exercises. This loss led to an increase in her symptoms, and she planned to make a new effort with the training:

My biggest problem was that I got pretty good results, so I stopped doing them...So now the problems have come back again. But I just need to start over.

#### Visualizing Treatment

The participants described the app as easy to use and having few technical problems. The training program gave them a fixed structure to follow, and they could individualize how fast they wanted to proceed in the program. They particularly appreciated the way the app visualized the training by offering a moving graph for each contraction. This visual helped them see for how long and at what intensity they should contract and relax the muscles:

Being able to see, partly to read the short intro and also see the picture properly, well yes, it just gives a completely different dimension in solving the problem.

Although the participants could memorize some of the exercises, after a while, they mostly chose to look at the app when performing them and reported that it helped them stay focused on the exercise.

### Good Enough?

#### Unsure Expectations of the Result

Despite high expectations from an app that supports training, the participants were unsure of their expectations about the actual treatment effect. They were “giving it a chance” or at least getting a little bit better with the app:

I probably expected that after three months I would be able to feel like I had done everything I could. I did not expect to feel like, oh, now I’m going to be cured for the rest of my life, but to know that I had at least given it an honest chance.

Some argued that it was difficult to know whether the result was good enough or if they could get even better if they exercised more:

It has become much better. And that makes me wonder about something, how good can it get actually?” Can I actually become, like, really, really good?

Some raised questions about how to evaluate or measure muscle strength:

And how good are my muscles, how well have I trained them? You never know that, I don’t think you ever get the answer sheet on that. Compared with others, or compared with people that don’t have leakage. Are their muscles stronger than mine, and things like that?

Some of the participants who did not get better after 3 months expressed that they would have liked to talk to someone about their results or lack thereof. They raised questions about what results could be expected, if they could get better by training for another 3 months, and where they should go if they wanted further help. At the same time, they felt satisfied that they had done what they could and felt that it helped them in their future decisions about whether to seek further help.

#### Insecurity About Training Correctly

After the treatment period, most participants felt confident that they could perform pelvic floor muscle contractions, had better control over their muscles, and had improved their muscle strength. At the same time, they were unsure about whether they could have been even stronger if they had contracted their muscles “better” or in a more “correct” way:

But I feel like I have found my muscles, but that I’m still a little bit uncertain about it, kind of...But you never know of course, would I have been even better if I had found my muscle groups better.

Although some participants would have liked someone to confirm that they were contracting correctly, most felt that the instructions within the app and their own experience performing PFMT were sufficient for them to feel confident that they did it right.

Well, I seem to remember that there was some text that said that in the beginning, you could feel for yourself to see if you were contracting in the right way, and I think that was good enough.

Participants who felt that their incontinence symptoms decreased considered this decrease a confirmation of the fact that they had performed the training correctly.

No, it’s more that I’ve tested it out and feel that I’m becoming successively stronger and stronger, so I have gotten that confirmation, that this was something important. Of course, the support in the instructions, what I would experience, that I would feel it dropping, feel it relaxing, and that’s exactly what I felt.

## Discussion

### Principal Findings

This qualitative study explored women’s experiences using an app-based treatment for SUI. We found that their experiences could be grouped into three categories. When deciding to try PFMT to treat their incontinence, the participants wanted some kind of support, and they searched for something new and something better than what they had previously tried, which is described in the category “Something new!” They also needed some help with motivation and coaching to be able to carry through the treatment they had engaged in, which is described in the category “Keeping motivation up!” In addition, they had to deal with the fact that performing a treatment independently involves a certain amount of uncertainty, which is described in the category “Good enough?” The core category “Enabling my independence” was related to all other categories and illustrates the women’s wish to take responsibility for their health and treatment.

### Core Category: Enabling My Independence

Our participants wanted to improve their health by self-treatment and thought that an app could be helpful. They had positive attitudes toward apps and were motivated to engage with them. This finding is in line with conclusions from a review by O’Connor et al, which showed that people who want to be healthier or have more control over their well-being are more likely to engage with digital health interventions [21].

Our core category summarizes the participants’ wish to manage their incontinence and PFMT independently and the ways the app could support them in achieving this. Similar findings have been described in a qualitative study on a mobile app to increase physical activity, where the participants appreciated the opportunity to use the app independently and described a feeling of being in control [22]. In addition, in a 2017 review of perceptions of health-tracking apps for chronic illnesses, the authors found that apps were an important support for managing the chronic illness, and users felt empowered to independently manage their illness [14].

### Providing Treatment Using an App

Our participants compared the app-based treatment to other self-management programs for SUI that they had tried before. They perceived the app as a more modern and effective method. It also seemed that the chance to use new technology made them feel like incontinence was prioritized. This finding is interesting and might be specific to an app dealing with a health condition considered to be “embarrassing” or “taboo.” As in our previous study on internet-based treatment, some women reported that participating in the study made it easier to bring up the otherwise sensitive topic of incontinence with friends and family [23]. Moreover, in this case, it is possible that the use of a new, modern, and “fun” tool like an internet program or a training app facilitated a willingness to “open up” the subject.

Our participants emphasized the convenience of using apps, which has been well described in previous studies [24,25]. Anderson et al found that convenience was the main reason people engaged with health apps [24]. The easy availability makes interaction, recording, and reminders easy. Our participants also found it less time consuming and embarrassing to use an app for advice about incontinence compared to booking appointments in conventional health care.

The participants described many barriers to performing regular PFMT, and by using an app, they hoped to overcome some of these barriers. The app was seen as a tool to put good intentions into practice. The app supported them in several ways—by its mere presence on their mobile phone, by providing a fixed structure for their training, and by reminding them to adhere to that structure.

Forgetting to practice is a common problem in the self-management of SUI, and reminders could be important for adherence [26]. A study of apps for nutrition behavior change for weight management found mixed perspectives on reminders [27]. Some participants found them useful, but others found them annoying, for example, if they arrived at inappropriate times or were not discreet enough. Our participants expressed little concern about this aspect, and one reason could be that they could individualize the reminders within the app to times that were convenient. The ability to personalize or customize app features is known to motivate app use [12]. Additionally, the reminders in our study could be described as “cues to action,” whereas in the study on apps for weight management, the reminders appeared after a person had forgotten to record something in the app.

The app helped visualize or “to see” the training, with the moving graphs showing each contraction. The participants continued to use this visual help even when they had memorized the exercise, because they found it helpful for staying focused. In a study on mobile apps for young people with type 1 diabetes [28], an important finding was that the app provided a new visual understanding of diabetes self-management. All the adolescent participants in that study [28] used the verb “to see,” and interestingly, our participants also commonly used this exact expression. Thus, it seems that visual support/visualization could be helpful for different populations and different diseases and is therefore likely to be of general importance.

Some participants reported that their motivation declined during the 3-month study period. One reason was getting bored when the novelty of the app had faded. We also found that some participants lost their motivation to continue when their incontinence improved, whereas some lost motivation for the opposite reason—lack of improvement. Both scenarios have been previously described with apps for other health conditions such as pain management and nutrition behavior change for weight management [24,27].

### Performing Pelvic Floor Muscle Training

An interesting finding is that most participants found PFMT difficult and challenging, but at the same time, they felt confident that they could master it well on their own. Some did not feel completely sure that they were doing the pelvic floor muscle contractions correctly, even though they could feel that their pelvic floor muscle strength improved and their incontinence symptoms reduced.

Difficulties in understanding how to do the exercises and knowledge of whether they are done correctly are known barriers to adherence to PFMT. Enablers related to these difficulties are feedback, affirmation of progress, and professional involvement [29]. In the context of self-management with the help of an app, there are, of course, limitations to what kind of feedback is possible. Nevertheless, most women can correctly perform pelvic floor muscle contractions after a simple verbal cue [30]. There was information within the app explaining how to find the “right” pelvic floor muscles and how to evaluate progress. Most participants found that the information within the app and their own experience made them confident that their pelvic floor muscle contractions were “good enough.” The additional feeling of uncertainty was possibly unrelated to a lack of knowledge or skills for performing PFMT but rather related to a lack of reassurance, which might be an inevitable aspect of self-management treatment. Participants who did not experience improvement in symptoms where naturally more likely to wish for personalized feedback.

One explanation for why some participants had difficulties evaluating their results could be that they had unclear expectations about what results to expect with their incontinence symptoms when entering the study. Goal setting plays a role in promoting adherence to PFMT, as described in a report on adherence strategies [31]. Similarly, in a study of experiences with behavioral interventions for urinary incontinence, such as PFMT, French et al found that factors that enabled clients to adhere to PFMT were having realistic goals and expectations [29]. Our app did not include an individualized goal-setting feature. However, we encouraged participants to follow the treatment program and practice PFMT three times daily for 3 months, which could be considered a training goal.

### Further Research and Implications for Practice

Further research on app features that can increase adherence to app-based treatments would be valuable, such as ways to encourage goal setting for people using apps for self-management. In addition, ways to increase motivation to use apps in the long-term would be of interest. The impact of more advanced features within apps, like more individualized information and feedback, competition, or social sharing, could be evaluated, bearing in mind that more complex apps might lose some of their “ease of use.”

The app Tät was designed for sustained use among women with SUI. The idea of using the app for more periodic treatment when the symptoms (and the motivation to reduce symptoms) increase is interesting. Periodic use is probably common with self-management tools like apps. Although evaluated as a stand-alone treatment program, in clinical practice, the app could be used as a complement to other treatments such as supervised PFMT within conventional health care.

The Tät app was designed as a first-line, conservative treatment program for SUI with a focus on PFMT. We have evaluated the app with its different components as a whole, but we could not determine which parts of the program have been effective. This qualitative study has provided more insight into which parts of the app treatment women appreciated and which parts they found difficult. This information has already led us to make improvements in the app, such as adding audio sounds to the exercises to simplify the training and including a calendar function to facilitate self-monitoring of the number of exercises performed. After the RCT was completed, the app was released for free in the App Store and Google Play in Swedish, English, German, Spanish, Arabic, and Finnish. To date, 54,000 people have downloaded the app and answered our short questionnaire. The app is CE (Conformité Européenne) marked as a Class 1 medical device in accordance with Swedish regulation LVFS 2003:11 [32]. We are following the use of the app to learn more about who uses it and how well it works outside a study setting.

### Strengths and Limitations

This is the first qualitative study of an app-based treatment for SUI. We considered this interview study an important complement to our RCT, because we wanted to understand how and why an app treatment could be effective for SUI. Grounded theory was a suitable method of analysis, because little previous knowledge is available about this subject and we could not know in advance what findings to expect.

One strength was that our participants used the app for a long time (3 months). This duration made it possible to explore attitudes towards app interventions, factors that affected motivation to use the app, and challenges with long-term adherence.

The same interviewer performed all the interviews and did a majority of the transcription, thereby obtaining a thorough understanding of the material. Telephone interviews have some obvious limitations such as the lack of body language and the challenge of achieving personal contact without seeing the other person. This difficulty might be one reason for the relatively short interviews. On the other hand, the method made it possible to interview participants from all over Sweden.

The interviewer and all coauthors are family physicians, which could be an advantage, since we are used to talking about urinary incontinence with our patients. On the other hand, we might have preconceptions about what constitutes the best treatment method for women with incontinence.

We purposefully selected participants to represent a broad range of experiences, with variations in age and residence. The educational level was overall high in our RCT, limiting transferability to other groups of women with other educational backgrounds. However, educational level has not been found to affect the ability to learn or perform correct PFMT contractions [30]. The high educational level might also reflect the fact that use of health apps is still more common among individuals with higher education [33].

### Conclusions

Women using an app-based treatment program for SUI reported that the app treatment enabled them to self-manage an up-to-date treatment for their condition. They perceived the app as a new and modern tool that is useful for maintaining motivation when performing a PFMT program. Many women had doubts about whether their training results were good enough but accepted that some level of uncertainty is inevitable with this kind of independent self-treatment. The app is an appreciated way of providing first-line treatment to women with SUI.

## Abbreviations

CE: Conformité Européenne
PFMT: pelvic floor muscle training
SUI: stress urinary incontinence
UI: urinary incontinence
